# Emerging strategies to overcome ovarian cancer: advances in immunotherapy

**DOI:** 10.3389/fphar.2024.1490896

**Published:** 2024-11-05

**Authors:** Tatiana Massariol Pimenta, Josiany Carlos de Souza, Bárbara da Silva Martins, Solenny Maria Silva Butzene, José Matheus Simões Padilha, Milleny Ganho Marçal, Guilherme dos Santos Elias, Leticia Batista Azevedo Rangel

**Affiliations:** ^1^ Department of Pharmaceutical Sciences, Health Sciences Center, Federal University of Espírito Santo, Vitória, Espírito Santo, Brazil; ^2^ Biotechnology Program/RENORBIO, Health Sciences Center, Federal University of Espírito Santo, Vitória, Espírito Santo, Brazil; ^3^ Biochemistry Program, Health Sciences Center, Federal University of Espírito Santo, Vitória, Espírito Santo, Brazil

**Keywords:** ovarian cancer, immunotherapy, cancer vaccines, CAR-T cell therapy, antibody therapy

## Abstract

Ovarian cancer is the second most common malignant neoplasm of gynecological origin and the leading cause of death from cancer in the female reproductive system worldwide. This scenario is largely due to late diagnoses, often in advanced stages, and the development of chemoresistance by cancer cells. These challenges highlight the need for alternative treatments, with immunotherapy being a promising option. Cancer immunotherapy involves triggering an anti-tumor immune response and developing immunological memory to eliminate malignant cells, prevent recurrence, and inhibit metastasis. Some ongoing research investigate potentially immunological advancements in the field of cancer vaccines, immune checkpoint blockade, CAR-T cell, and other strategies.

## 1 Introduction to ovarian cancer immunotherapy

Ovarian cancer (OC) ranks first among deaths caused by gynecological malignant neoplasms around the world (American Cancer Society, 2024). OC’s dramatic epidemiological scenario is related to diagnoses in advanced stages of the disease, due to the absence of pathognomonic signs and symptoms for early diagnosis (Doubeni et al., 2016), coupled with the first-choice therapeutic regimens chemoresistance acquisition by OC cells (Ghoneum et al., 2021). These conditions require other ways to treat these patients, other than surgeries and non-specific conventional chemotherapy. In consequence, different immunotherapy approaches have arisen as relevant alternatives to overcome this treatment obstacle (Bund et al., 2022).

OC immunotherapy involves the induction of an anti-tumor immune response and the development of immunological memory. This process not only can eradicate malignant cells within the primary tumor site, thereby averting recurrence, but also hampers the metastatic spread to distant anatomical locations (Cha et al., 2020). Presently, the Food and Drugs Administration (FDA) has sanctioned some distinct immunotherapeutic modalities for OC or is actively investigating them in clinical trials (Cha et al., 2020). These approaches can be categorized into active and passive immunotherapies.

Active immunotherapy harnesses the immune system to identify and target specific cancer antigens. It includes vaccines that stimulate the patient’s immune response, or chimeric antigen receptor (CAR) T-cell therapy, which involves the reintroduction of genetically engineered T-cells in the patient (Rui et al., 2023). On the other hand, passive immunotherapy modulates the activity of a patient’s immune system response, as observed with immune checkpoint inhibitors (ICIs) molecules (Rui et al., 2023). In this review, we compile the latest findings concerning OC immunotherapy strategies.

## 2 Therapeutic OC vaccines

To handle the adverse effects of common therapies for cancer, immunotherapy strategies emerged as a cancer-specific alternative capable of targeting the tumor and causing minimal impact on normal tissues (Aly, 2012; Zhu and Yu, 2022). They are significant considering the usual therapeutic approaches such as surgery, chemotherapy, and radiotherapy which besides the adverse effects show a lack of specificity for tumors (Kaczmarek et al., 2023). Therapeutic cancer vaccination is a strategy of immunotherapy developed to elicit or boost antitumor adaptive immune responses to detect and eliminate them (Luo, et al., 2024; Chambers, 2011). This response is specifically direct against malignant cells leading to the inhibition of tumor growth and/or recurrence (Siminiak et al., 2022). Cancer vaccines use diverse mechanisms to provoke the immune system and develop a specific anti-tumor response (Shafabakhsh et al., 2019; American Cancer Society, 2020) and immunological memory that may prevent recurrences (Janes et al., 2024).

**FIGURE 1:**
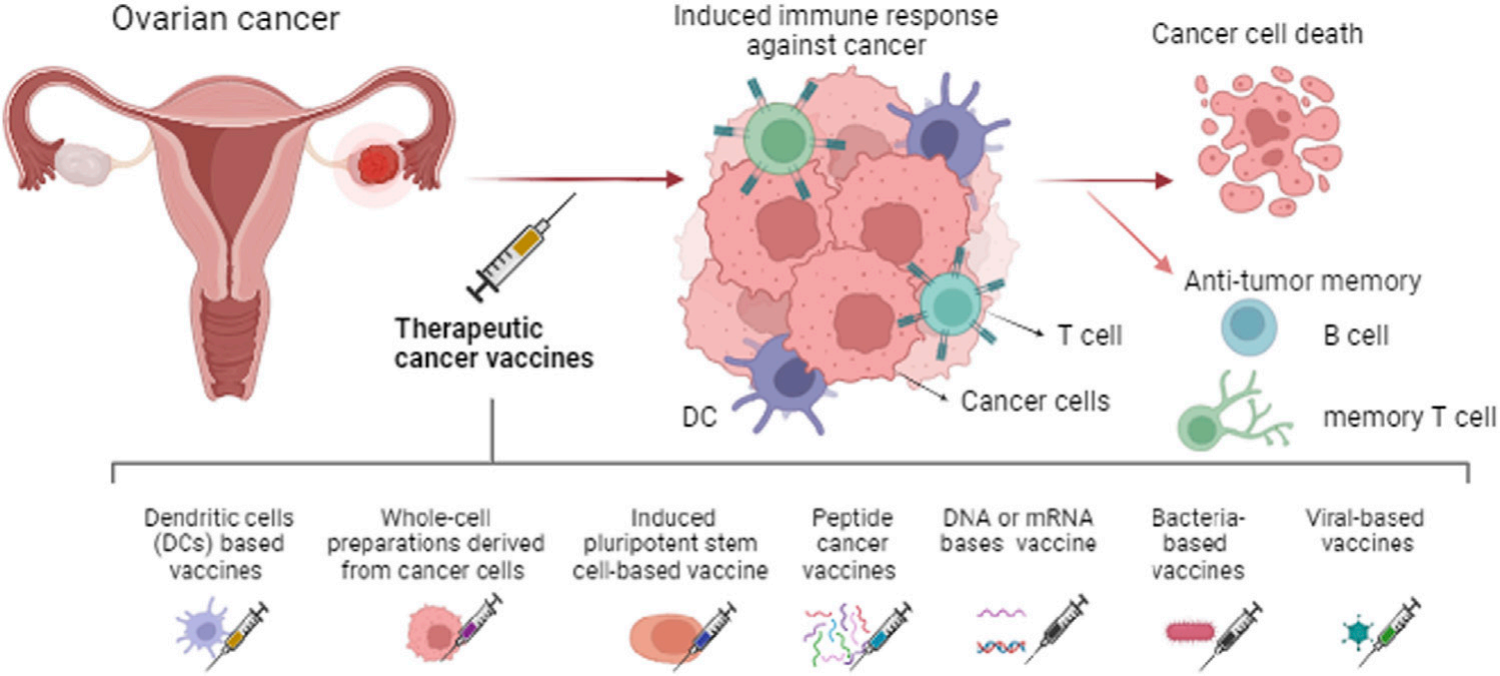
Types of therapeutic cancer vaccines and the main mechanism induced. The immune system response is generated against the tumor leading to cancer cell death and immunological memory.

OC, which is a challenging disease to diagnose and treat, usually shows resistance to available chemotherapies and frequently relapses with more aggressiveness (Acharya et al., 2024). The clinical characteristics demonstrate the importance of developing novel therapeutic strategies to treat and overcome chemoresistance in OC. In this scenario, different cancer vaccines have been studied in OC. The main mechanisms of cancer vaccines involve the induction of dendritic cells (DCs) potent antigen-presenting cells (APCs), these cells identify and present the antigen for other cells using major histocompatibility complex (MHC) molecules (Lin M. et al., 2022). Also secrete IL-10, IL-12, IL-23, and TNF-β to stimulate the differentiation of immune system cells (Zhang X. et al., 2021). CD8^+^ cytotoxic T lymphocytes (CTLs) recognize the antigens presented on MHC class I molecules, leading to their activation and proliferation, consequently, attacking and destroying the tumor (Kaczmarek et al., 2023). CD4^+^ helper T cells recognize peptides presented on MHC class II molecules and provide support to other immune cells. B cells can also be activated resulting in the production of antibodies specific to the tumor-associated antigens (TAAs) (Janes et al., 2024). These antibodies can directly bind to tumor cells, aiding in their destruction. The vaccine also aims to induce a memory response, which enhances immune protection and provides a more robust response upon future encounters with tumor cells expressing the same TAAs (Fan et al., 2023), see Figure 1.

DCs based vaccines depend on *ex vivo* modification of DCs from the patient or cells created in the laboratory. Immune-stimulating agents or tumor-specific antigens (TSAs) obtained from tumor cells or genetic material are applied to mature and activate these cells followed by reinfusion into the patient. Once reinfused, these cells interact with T cells, B cells, and natural killer (NK) cells (Lv et al., 2020; Fan et al., 2023). The activation of immune system cells, as mentioned above, enhances the immune response and destroys cancer cells (Laureano et al., 2022). The use of this kind of vaccine has shown relevant results, for example, a study using an autologous dendritic cell-based vaccine with tumor lysate after systemic chemotherapy resulted in a decrease in progression rate, as well as improved overall survival in OC (Zhang X. et al., 2021). A Th17-inducing folate receptor alpha (FRα)-loaded DCs vaccine, resulted in the development of Th1, Th17, and antibody responses to FRα in most patients. These processes are associated with prolonged recurrence-free survival and induce antigen-specific immunity (Block et al., 2020). Another approach combined a whole tumor lysate-pulsed dendritic cell vaccine with bevacizumab, cyclophosphamide, aspirin, and interleukin-2, this vaccine produced T-cell responses and was associated with increased overall survival of patients (Tanyi et al., 2021).

A similar mechanism is induced by the whole-cell preparations or lysates derived from cancer cells reintroduced into the patient (Chiang et al., 2011). Cells are sourced from the patient’s tumor or established cancer cell lines, aiming to prevent their growth and pathogenicity the cells are inactivated or genetically modified (Kaczmarek et al., 2023; Pérez-Baños et al., 2023). Another approach utilizes induced pluripotent stem cell (iPSC)-based cancer vaccines. iPSCs are created from somatic cells and then differentiated into tumor microenvironment (TME)-specific cells, such as tumor-associated fibroblasts, endothelial cells, or immune cells (Chehelgerdi et al., 2023). These iPSC-derived cells express antigens characteristic of the TME, including TSAs or molecules associated with immunosuppression. When administered to the patient, these cells are recognized by immune cells, triggering a robust immune response (Ouyang et al., 2019). Zhang Z. et al. (2012) used human embryonic stem cells as a OC prevention vaccine in rats, this vaccine caused anti-tumor responses and enhanced tumor rejection in the animal models.

Peptide cancer vaccines are also an emerging treatment for OC, using specific epitope peptides derived from TAAs or TSAs (Abd-Aziz and Poh, 2022). This vaccine can stimulate the immune system after being administered and taked up by APCs (Wada et al., 2016; Liu et al., 2024a). Recent studies in phase I or II use mutated p53 peptides (The cancer-testis antigen, named New York esophageal squamous cell carcinoma-1, NY-ESO-1) and also apply diverse technologies to treat OC in association with co-therapies (Odunsi, 2017; Siminiak et al., 2022). Vaccines made from a peptide or antigen may help the body build an effective immune response to kill tumor cells, functioning as a booster for the patient’s anti-tumor immune response and the combination with chemotherapy may induce the death of more tumor cells (Bund et al., 2022; Odunsi, 2017).

In a phase I/IIa trial (Brown et al., 2019) used E39 in patients HLA-A2+, this is an immunogenic peptide derived from the folate-binding protein, frequently found overexpressed in multiple malignancies. When associated with granulocyte macrophage-colony stimulating factor (GM-CSF) was able to improve disease-free survival (DFS) of endometrial cancer and OC patients (90.0% vs. Control Group: 42.9%). Targeting folate receptor (FR) a vaccine was tested in patients with OC or breast cancer. The vaccine stimulated or increased immunity in more than 90% of patients and the FR T cell responses were detectable for at least 12 months. The results demonstrate the benefits of boosting immunity to tumors expressing FR antigen (Kalli et al., 2018). O’Cearbhaill et al. (2019) combined a polyvalent vaccine conjugate responsible for inducing antibody responses (Globo-H, GM2, MUC1-TN, TF) with adjuvant OPT-821 in patients with OC in remission after chemotherapy. Vaccine + OPT-821 compared to OPT-821 alone was modestly more immunogenic.

Cancer vaccines can also involve genetic material (DNA and RNA) encoding TAAs. This DNA or RNA is taken up by cells, such as DCs, and the TAAs are presented on the surface of APCs after being processed. In this process, the activation and proliferation of CD8^+^ CTLs are induced and CD4^+^ helper T cells provide support to other immune cells (Pandya et al., 2023). Additionally, B cells can be activated by presented TAAs and induce the production of antibodies. These antibodies can bind directly to tumor cells, aiding in their destruction (Barbier et al., 2022). The vaccine also aims to induce a memory response, which enhances immune protection and ensures a more effective response upon future encounters with tumor cells expressing the same TAAs (Wang B. et al., 2023a). Lu et al. (2023) using immuno-bioinformatics developed a model of a multi-epitope mRNA self-adjuvant vaccine targeting CA-125 neoantigen in breast and ovarian cancers. This *in silico* analysis provided evidence of using this neoantigen in a mRNA-based vaccine. Posity results were observed using the SynCon FSHR DNA vaccine. In this study synthetic consensus (SynCon) approach was capable of breaking immune tolerance to follicle-stimulating hormone receptor (FSHR). The treatment induced robust CD8^+^ and CD4^+^ cellular immune responses and FSHR-redirected antibodies in mice, as well, delayed the progression of aggressive OC model with peritoneal carcinomatosis (Perales-Puchalt et al., 2019).

Neoantigen DNA vaccines were used by Bhojnagarwala et al. (2021) to target ∼40 neoantigens. These plasmid-based vaccines were able to provoke long-term immune responses against lung and ovarian cancer and protected animals from tumor growth for 89 days after the final vaccination. Another DNA vaccine platform targeting tumor neoantigens was applied against lung and ovarian cancers affecting the tumor progression and survival in mouse models. In this pre-clinical study, the vaccine was able to generate potent CD8^+^ T-cell antitumor–specific responses *in vivo*. Interestingly, when neoantigen-specific T cells were expanded from immunized mice they were also able to kill tumor cells *ex vivo* (Duperret et al., 2019).

Bacteria-based cancer vaccines use engineered bacteria to stimulate the immune system (Zhou et al., 2023). These modified bacteria interact with immune cells, initiating an inflammatory response and triggering the production of pro-inflammatory cytokines, chemokines, and other signaling molecules (Zalatan et al., 2024). Viral-based cancer vaccines use engineered viruses to stimulate the immune response directly. These modified viruses interact with immune cells such as DCs, macrophages, and NK cells, triggering an inflammatory response along with the release of pro-inflammatory cytokines and chemokines (Xu et al., 2024). Immune cells then phagocytose the virus particles, and TAAs expressed by the virus or introduced into infected cells are processed and presented to T cells (Muthukutty and Yoo, 2023). Cowpea mosaic virus co-delivered with irradiated OC cells comprises an prophylactic vaccine against a model of OC in mice. After two vaccinations most of the mice (72%) reject the tumor challenges, and survived subsequent rechallenges, indicating immunologic memory (Stump et al., 2021).

These approaches highlight the diverse strategies being employed to develop effective vaccines for OC, with ongoing research focused on optimizing these therapies and evaluating their clinical efficacy. The actual scenario for cancer vaccines is due to years of research and discoveries. Nevertheless, the heterogeneity of the immune system and the capacity of cancer cells to evade immune system attacks, even when naturally endogenous or when induced by vaccine makes this process a challenge. This is why more in-depth studies must be completed to enable the large use of these therapies.

## 3 CAR-T cell therapy in OC

CAR-T cells are genetically engineered to recognize and attack TSAs (June et al., 2018), bypassing the need of MHC molecules presentation, and behaving as active drugs against tumors (Maus and June, 2016). FDA approved CAR-T therapy in 2017 (reviewed by Yi-Ju et al., 2023), with two treatments, Yescarta (axicabtagene ciloleucel) and Kymriah (tisagenlecleucel), specifically for certain lymphomas and leukemia (Food and Drug Administration, 2024). Despite its clinical success in treating blood cancers, CAR-T therapy can lead to serious complications (reviewed by Brudno and Kochenderfer, 2024). These include cytokine release syndrome (CRS), which can cause extreme symptoms like high fevers, organ failure, and even death (Reagan and Neelapu, 2021). Another risk is “on-target, off-tumor toxicity,” where CAR-T cells attack healthy tissues, causing severe harm (Flugel et al., 2023). Additionally, the required lymphodepleting chemotherapy before CAR-T infusion is genotoxic, raising the risk of secondary cancers and other diseases (Yeh et al., 2020). Since then, extensive global research has been conducted on various hematologic and solid tumors to evaluate the safety and efficacy of CAR-T therapy and it has shown significant success in treating hematologic cancers, with six other FDA approvals, and holds promise as a new treatment option for OC (reviewed by Cappell and Kochenderfer, 2023).

Solid tumors present significant challenges for CAR-T cell therapy due to their heterogeneity and the scarcity of known tumor-specific epitopes (Labanieh and Mackall, 2023). Unlike hematological malignancies, solid tumors often result in toxicity when targeting overexpressed antigens (reviewed by Baker et al., 2023). Additionally, the TME creates physical and immunological barriers that limit CAR-T cell effectiveness (reviewed by Albelda, 2024). To overcome these obstacles, researchers are exploring intratumoral injections (Tchou et al., 2017), peptide and nanoparticle booster vaccines (MA et al., 2019; Reinhard et al., 2020), engineered cytokine-driven expansion (Sockolosky et al., 2018), and modifying the TME with oncolytic viruses and genome editing techniques like CRISPR-Cas9 (reviewed by Baker et al., 2023).

Emerging clinical data show promise for CAR-T cells targeting solid tumors, including prostate cancer (prostate-specific membrane antigen) (Narayan et al., 2022), gastrointestinal cancer (CLDN18.2) (Qi et al., 2022), glioblastoma (IL13RA2 or EGFRv3) (Sampson et al., 2020), and neuroblastoma (GD2) (Del Bufalo et al., 2023). Despite these advances, challenges persist due to the scarcity of unique, tumor-specific targets (Macpherson et al., 2020). In OC, potential targets identified include mesothelin (MSLN) (Schuster et al., 2017), Muc16 (Coelho et al., 2018), TAG72 (Murad et al., 2018), FR (Rodriguez-Garcia et al., 2017), and FSHR (Perales-Puchalt et al., 2017). Furthermore, recent studies have explored the feasibility, safety, and anti-tumor activity of the first-in-human approach of targeting CLDN6 with CAR-T therapy and combining it with a CAR-amplifying vaccine (Mackensen et al., 2023), given the frequent detection of high-level CLDN6 in epithelial OC, endometrial carcinoma, and other solid tumors (Jaeger et al., 2014). Hence, CAR technology using NK cells is being studied for a range of solid tumors, as well as OC (reviewed by Dagher and Posey, 2023). Table 1 highlights some studies that evaluate CAR technology use in OC and other cancer types.

**TABLE 1:** Ongoing studies evaluating CAR technology in OC and other tumors treatments. Clinical trials that have recently started using CAR cell technology in OC are currently in “recruiting” status. Some CAR cells have undergone modifications to become more specific or to avoid some side effects, such as CRS.

CAR technology	Modification	Target	Clinical study phase	References
CAR T cell - iC9-CAR.B7-H3	Presence of an inducible suicide gene, caspase 9 (iC9). CAR T cells are eliminated in a severe CRS event	B7-H3 Immune checkpoint most expressed in tumors, associated with poor prognosis	I	NCT06305299 Miyamoto et al. (2022)
CAR T cell - 27T51	Presence of an anti-MUC16 site	MUC-16 Antigen commonly expressed in OC. Increased efficacy *in vivo*	Ia/Ib	NCT06469281 Chekmasova et al. (2010)
CAR T cell	CAR T cells specific for Cluster of differentiation 70 (CD70)	CD70 Glycoprotein related to chemoresistance in OC.	I	NCT06215950 NCT06383507 NCT06010875 Aggarwal et al. (2009)
CAR T cell Tmod™	Activation in presence of MSLN. Addition of HLA-A*02 inhibitor	Tumors that express second-generation MSLN and have lost HLA-A*02 expression. Associated to poor prognosis	I and II	NCT06051695 Andersson et al. (2012) Tokatlian et al. (2022)
CAR-iNK cell (FT536)	Affinity for MICA and MICB. IL-15 expression (improves the complex performance)	MICA and MICB (overexpressed in OC)	I	NCT06342986 Li K. et al. (2009) Lee D. et al. (2023)
CAR-iNK cell	Umbilical cord blood-derived NK cells transduced with IL-15 and engineered with CAR TROP2	TROP2 Overexpressed protein and associated with proliferation and invasion in OC.	I and II	NCT05922930 Wu et al. (2017)
CAR-iNK cell - SynKIR-110	Presence of a killer cell immunoglobulin-like receptor (KIR)	MSLN. Glycoprotein commonly overexpressed in OC and associated with tumor progression	I	NCT 05568680 NCT06256055 Liang et al. (2021) Hilliard (2018)

## 4 Exosomes in OC treatment

Exosomes represent a promising tool and target for immunotherapy in OC (Zhou W. et al., 2021). Although they are physiological components, their role in cancer remains somewhat ambiguous. In the context of immunotherapy, these lipophilic vesicles are crucial for facilitating communication among immune system cells, which can either elicit positive immune responses or lead to immunosuppression (Kugeratski and Raghu, 2021). Taylor et al. (2003) demonstrated that membrane fragments, which include exosomes and other lipid vesicles, derived from OC cells can induce T cell apoptosis. The influence of exosomes and similar membrane fragments on orchestrating immune system responses has been explored in various cancer types, including breast (Morrissey et al., 2021), lung (Alipoor et al., 2018), pancreatic (Shen et al., 2020), glioma (Li M. et al., 2022), and colorectal cancer (Zhao S. et al., 2020). Consequently, several key aspects regarding the role of exosomes in immunotherapy will be discussed below.

Exosomes are a category of extracellular vesicles with a lipid bilayer, measuring approximately 30–150 nm, found in various body fluids such as blood, urine, saliva, and cerebrospinal fluid (He et al., 2018; Gong et al., 2023). They are believed to have a dual role in the TME (Li X. and Wang, 2017). Exosomes can both promote and inhibit tumors and carry many potential biomarkers for OC (Gong et al., 2023). In normal cells, these small vesicles can interact with membrane receptors or fuse with cells to release components such as proteins, RNA, DNA, mRNA, miRNA, long non-coding RNA (lncRNA), and lipids, aiding in cellular communication, extracellular matrix maintenance, and immune system modulation (Pegtel and Gould, 2019; Kaushik and Cuervo, 2015; Ramirez and Marcilla, 2021; Zhu et al., 2024; Tian et al., 2022). In cancer cells, exosomes perform similar functions but carry components that promote proliferation, migration, invasion, chemoresistance, and other processes that enhance malignancy, complicating treatment, such as modulation of the TME (Bhattacharya et al., 2024; Yim et al., 2020; Li X. et al., 2021b).

In OC, exosomes play a dual role in the acquisition of chemoresistance, a process caused by the lack of cancer cells response to chemotherapy, often resulting in treatment failure (Tian et al., 2022; Liu H. et al., 2024b; Carmi et al., 2024). In this context, their malignant role in OC was elucidated by Pan et al. (2024). Their study found that exosomes derived from OC stem cells were responsible for increasing chemoresistance and proliferation while inhibiting apoptosis in the cisplatin-resistant SKOV3 cell line. Meanwhile, exosomes derived from ascites were observed to carry a lncRNA that sensitized high-grade serous ovarian cancer (HGSOC) cells to cisplatin chemotherapy, a standard drug for this OC subtype. Additionally, it was demonstrated that exosomes carried a lncRNA that reduced cell proliferation, migration, and invasion in both *in vitro* and *in vivo* experiments (Liu H. et al., 2024b).

Another factor complicating chemotherapy treatment is the low oxygenation within tumors, resulting from reduced blood perfusion. Wang Q. et al. (2024) analyzed this process and observed that tumor-derived exosomes contributed, in part, to the decreased oxygenation through the previously mentioned mechanism, by altering the tumor vascular network and thereby impeding chemotherapy.

**FIGURE 2:**
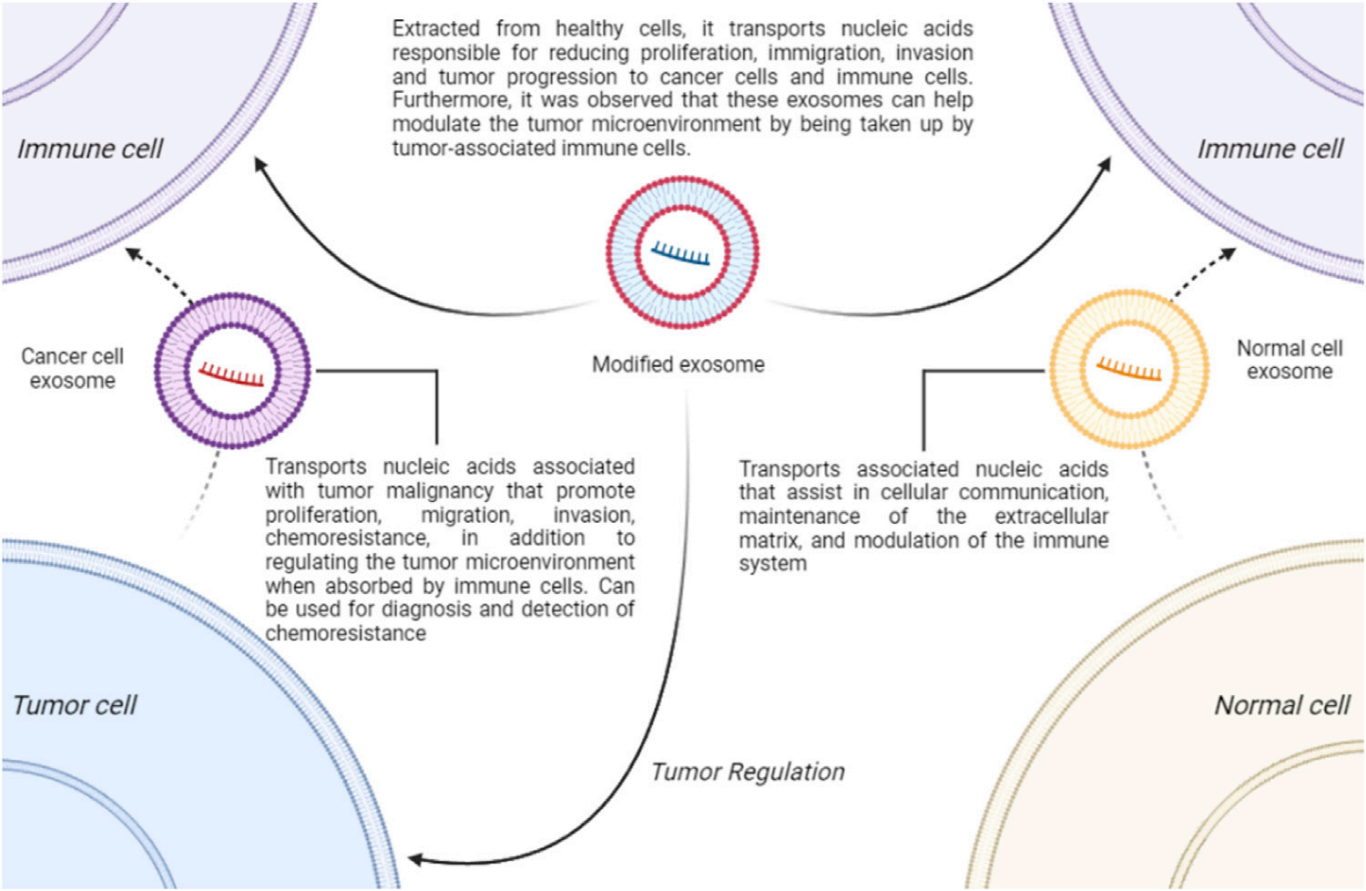
The Role of Exosomes. Exosomes play a physiological role in cellular communication, immune system modulation, and maintenance of the extracellular matrix. In OC, they are associated with tumor progression, proliferation, migration, invasion, and regulation of TME. Tumor cell-derived exosomes can serve as biomarkers for diagnosis and early detection of chemoresistance. Additionally, modified exosomes, such as those derived from hUC-MSCs or engineered using nanotechnology, may aid in treatment by reducing tumor progression and potentially modulating the TME.

In addition to their ambiguous role, exosomes may serve as a potential tool for OC therapy, as demonstrated in the study by Shimizu et al. (2024). In this study, exosomes were extracted from a cell culture of fibroblasts from OC patients and were loaded with siRNAs targeting a proto-oncogene, the MET receptor. This treatment inhibited OC cells proliferation, migration, and invasion. Another study showed that it is possible to create targeted exosomes for OC treatment (Mousaei Ghasroldasht et al., 2024). Mousaei Ghasroldasht et al. (2024) developed what they termed “enhanced exosomes” using a culture of human umbilical cord-derived mesenchymal stem cells (hUC-MSCs), observing that these exosomes contained proteins and miRNAs capable of regulating and sensitizing OC. Another study, by Kim et al. (2023), used a nanotechnology-modified exosome in glioma to evaluate its effectiveness. The results indicate that there was regulation of the TME and decreased tumor progression both *in vitro* and *in vivo*. Furthermore, exosomes can be utilized as biomarkers for an improved and earlier diagnosis, addressing the delays often seen in most cases (Bhavsar et al., 2024; Zhu et al., 2024; Xiao et al., 2022). There is also evidence that these vesicles carry RNAs related to chemoresistance and, therefore, may serve as biomarkers for this process, which precedes clinical interventions (Asare-Werehene et al., 2020; Li T. et al., 2021a). These findings suggest that exosomes have intriguing therapeutic potential warranting further investigation. Therefore, deepening studies in this area is crucial to better understand the contribution of these components in OC immunotherapy and the underlying mechanisms of different kinds of exosomes and how they influence on tumor response to treatment (Figure 2).

## 5 Antibody-based therapies for OC treatment

Therapeutic monoclonal antibodies have been successfully developed for the treatment of various cancer types (Hafeez et al., 2020).

In this context, with the biotechnology advancement, antibody-drug conjugates (ADCs) have been developed, representing one of the newest classes of cancer medications, with approvals for the treatment of solid tumors as well as hematological malignancies. ADCs exhibit high selectivity for tumors, thereby minimizing their systemic exposure, which potentially leads to an improved therapeutic index, offering greater efficacy and fewer side effects (Dean et al., 2021). To minimize off-target toxicity, the target antigen should be exclusively or preferentially expressed in cancer cells, with minimal expression in healthy tissues (Hafeez et al., 2020). Several monoclonal antibody-based immunotherapies have already been approved by FDA (Zhou et al., 2023). However, numerous clinical trials are still underway with promising prospects for the treatment of OC, including ADCs such as JNJ-78306358, ivonescimab, ipilimumab, durvalumab, oregovomab, catumaxomab, abagovomab, daclizumab and mirvetuximab, which was approved by the FDA in 2022 but remains under study for application in OC treatment (Dilawari et al., 2023).

### 5.1 Immune checkpoint inhibitors (ICIs)

Cancer cells develop several complex mechanisms to evade the immune system in the TME, among which the inhibition of T cell activity by the PD-1/PD-L1 (Daud et al., 2016) and CTLA-4/B7 pathways can be highlighted (Tang Q. et al., 2022a). PD-1 is an immune receptor expressed on the surface of various immune cells, and the interaction between PD-1 and PD-L1, which is highly expressed on the surface of tumor cells and tumor-infiltrating cells, results in the inhibition of T cell activity, rendering the anti-tumor immune response ineffective and favoring immune evasion (Daud et al., 2016; Naimi et al., 2022; Tang Q. et al., 2022a). Furthermore, the binding of PD-1 to its ligand can inhibit T cell proliferation, B lymphocyte differentiation, and the production of cytokines such as Interferon-gamma (IFN-Y) (Tang S. et al., 2022b).

On the other hand, another immune checkpoint associated with tumor cell evasion is CTLA-4, an inhibitory receptor belonging to the immunoglobulin superfamily (Van Coillie et al., 2020). CTLA-4 is primarily expressed on activated T cells and, like PD-1, has an immunomodulatory function (Tang S. et al., 2022b). The interaction of CTLA-4 with its ligands, B7-1 (CD80) and B7-2 (CD86), expressed on APCs and tumor cells, transmits a signal that negatively regulates or interrupts T cell activity, thereby decreasing the immune response against cancer cells (Naimi et al., 2022; Tang S. et al., 2022b; Van Coillie et al., 2020).

From this perspective, ICIs represent a promising class of drugs in immunotherapy against OC, targeting PD-1/PD-L1 and CTLA-4. They have already demonstrated broad bioactivity and stable response in the treatment of various types of tumors (Naimi et al., 2022; Tang S. et al., 2022b), including OC (Disis et al., 2019).

#### 5.1.1 PD-1/PD-L1 inhibitors

Recent studies conducted by Friedman et al. (2024) involving 35 patients demonstrated that the use of nivolumab, a PD-1 inhibitory monoclonal antibody, in the treatment of uterine cancer and OC with DNA mismatch repair deficiency (dMMR) showed clinical efficacy with an objective response rate (ORR) of 57%. Additionally, 64.7% of patients experienced progression-free survival (PFS) at 24 weeks, and treatment toxicity was moderate. However, while the results are promising, further studies with a larger cohort representing the population of patients with OC-dMMR are necessary, as well as the identification of additional predictive biomarkers for treatment response and resistance.

On the other hand, another notable ICI is ivonescimab, also known as AK112 and SMT112. It is a humanized bispecific antibody whose single-chain variable fragments (ScFv) bind to the C-terminus of each anti-VEGF antibody heavy chain (Wang L. et al., 2023b), forming a complex with high affinity for PD-1 (Zhao et al., 2023). Ivonescimab is currently being evaluated in clinical studies for its anti-PD-1 and anti-VEGF-A activities, with the goal of preventing tumor progression through the inhibition of angiogenesis (Apte et al., 2019). The anticipated outcomes of this inhibition include reduced immunosuppression and decreased tumor angiogenesis (Dhillon, 2024). However, clinical trials have encountered challenges in achieving satisfactory results.

In the phase Ia study by Frentzas et al. (2024), the activity of ivonescimab was evaluated in 19 patients with platinum-resistant OC. Among these patients, 68.4% had received more than three lines of prior therapy. Of the 19 patients, five achieved a partial response, including 3 with high-grade serous pathology and 2 with clear cell pathology, resulting in an ORR of 26.3%. Additionally, the study observed that the disease remained stable for more than 12 months in four patients who had previously been treated with bevacizumab. However, further clinical studies are needed to determine more appropriate dosages and to conduct additional analyses in combination therapies.

#### 5.1.2 CTLA-4 inhibitors

One of the promising antibody-drugs in this class is ipilimumab, a monoclonal antibody targeting CTLA-4 (Saad and Kasi, 2023). Knisely et al. (2024) conducted a phase Ib study evaluating intraperitoneal ipilimumab and nivolumab in patients with recurrent gynecological neoplasms with peritoneal carcinomatosis. The study included 23 patients: 18 with OC, 2 with uterine cancer, and 3 with cervical cancer. In this study, a partial response was observed in two patients (8.7%), one with OC and one with uterine cancer, with a response duration of 14.8 months. Additionally, the treatment safety was assessed, revealing that two patients (8.7%) experienced adverse effects classified as grade 3 or higher. Despite these adverse effects, the study found that treatment with ipilimumab and nivolumab can produce lasting responses in the treatment of OC.

#### 5.1.3 Combined therapies

Hinchcliff et al. (2024) conducted a phase II randomized clinical trial comparing durvalumab (PD-L1 inhibitory monoclonal antibody) and tremelimumab (anti-CTLA-4 antibody) administered either as a combination therapy or sequentially in patients with platinum-resistant OC. Among the patients, 38 received sequential therapy (tremelimumab followed by durvalumab), while 23 received combination therapy (tremelimumab and durvalumab together, followed by durvalumab alone). There was no significant difference in PFS between the combination therapy group (1.84 months) and the sequential therapy group (1.87 months) (*p* = 0.402). Partial responses were observed in two patients (8.7%) and stable disease in 1 patient (4.4%), with all responses occurring in the combination therapy group.

Landry et al. (2023) reported promising results from a phase Ib study investigating the combination of durvalumab with eribulin, a microtubule inhibitor with established benefits in metastatic breast cancer (MBC). The study included four patients with recurrent OC and five patients with HER2-negative MBC, all of whom received escalating doses of eribulin along with durvalumab. The results indicated an ORR of 55%, with four patients experiencing stable disease, and a PFS of 6.2 months.

On the other hand, Konstantinopoulos et al. (2019) demonstrated that the combination of niraparib, a PARP inhibitor (PARPi), with pembrolizumab (anti-PD-1 antibody) showed promising activity in the treatment of platinum-resistant recurrent OC patients. This combination resulted in reduced tumor size and observed disease stabilization. Furthermore, the study indicated that the combination enhanced treatment efficacy, achieving an ORR of 19%, compared to monotherapy with each agent. No new signs of toxicity were reported in this study. Hence, those studies suggest that the combination between ICIs with other drug classes may offer a viable alternative for improved treatment outcomes.

### 5.2 Antibody therapies using ADCs

#### 5.2.1 JNJ-78306358

It is well established that human leukocyte antigen G (HLA-G) is minimally expressed in healthy cells but highly expressed in various types of human cancer cells (Lin A. and Yan, 2018), including OC. HLA-G functions as an immune checkpoint and interacts with inhibitory receptors (Geva et al., 2024).

In this context, the phase I study by Geva et al. (2024) found that JNJ-78306358, an ADC that binds simultaneously to the α3 domain of HLA-G isoforms on tumor cells and the CD3 receptor complex on T cells, facilitated the formation of immune synapses and the killing of tumor cells by CTLs in renal cell carcinoma, OC, and colorectal cancer in 39 patients. Conversely, no interaction of this ADC was found with cells that do not express HLA-G, demonstrating its specificity for certain types of tumor cells. In this study, all 39 patients (100%) discontinued treatment. The most frequent reasons for discontinuation were disease progression (82.1%) and death (5.1%), with none attributed to the ADC JNJ-78306358.

#### 5.2.2 Mirvetuximab

Among the highly important and promising ADCs for OC treatment, mirvetuximab was approved by FDA in 2022, based on the results from the SORAYA study (Matulonis et al., 2023). This ADC consists of an IgG1 monoclonal antibody targeting the folate receptor alpha (FRα) conjugated to the cytotoxic maytansinoid DM4, which has demonstrated significant clinical activity in patients with FRα-positive OC (González-Ochoa et al., 2023).

Richardson et al. (2024) presented results from a phase Ib study combining mirvetuximab soravtansine with carboplatin and bevacizumab in patients with platinum-sensitive OC. In this study, 41 patients were enrolled, of whom 34 exhibited an anti-tumor response, resulting in an ORR of 83%. Most adverse effects were graded as two or lower, indicating an acceptable safety profile.

Another study involving mirvetuximab was conducted by Moore et al. (2023), who reported results from a global, phase III, confirmatory, open-label, randomized, and controlled trial for the treatment of platinum-resistant FRα-positive HGSOC. Among the patients, 227 were assigned to the mirvetuximab group and 226 to the chemotherapy group (paclitaxel, pegylated liposomal doxorubicin, or topotecan). The results showed a median PFS of 5.62 months and an ORR of 42.3% in the mirvetuximab group. During treatment, fewer grade 3 or higher adverse events occurred with mirvetuximab (41.7%) compared to chemotherapy (54.1%), as well as fewer serious adverse events of any grade (23.9% vs. 32.9%) and events leading to discontinuation (9.2% vs. 15.9%), demonstrating greater safety with the ADC treatment.

#### 5.2.3 Oregovomab

The ADC oregovomab is a murine monoclonal antibody that binds to cancer antigen-125 (CA-125) in blood and local tissues (Battaglia et al., 2020). It is administered to induce targeted therapeutic immunity against cancer. The oregovomab-CA125 complex has enhanced efficacy in antigen capture and cross-presentation, which activates cellular immune response (Brewer et al., 2020).

In this context, Brewer et al. (2020) conducted a phase II, international, randomized, multicenter study to evaluate the results of chemoimmunotherapy in OC using carboplatin-paclitaxel and indirect immunization with oregovomab. The study involved 94 patients who were randomly assigned to receive either carboplatin-paclitaxel alone or carboplatin-paclitaxel with oregovomab addition. Results showed that all patients achieved cytoreduction to less than 1 cm of residual disease or no macroscopic residual disease. Furthermore, the median PFS was 41.8 months in patients receiving additional oregovomab compared to 12.2 months in the control group, demonstrating a significant difference between the two groups (*p* = 0.0027).

Additionally, a multicenter phase II study by Park et al. (2024) examined the efficacy of non-platinum-based chemotherapy with the use of oregovomab in patients with recurrent OC. This study demonstrated promising efficacy, achieving a PFS of 11 weeks and a median overall survival of 70.4 weeks.

#### 5.2.4 Catumaxomab (Removab)

Catumaxomab is a trifunctional bispecific ADC and targets epithelial cell adhesion molecule (EpCAM) and CD3 T-cell antigen (Ruf et al., 2021). Its anti-tumor effect results from a complex immune reaction at the tumor site involving T cell-mediated lysis, which includes T cell-mediated destruction of tumor cells, antibody-dependent cellular cytotoxicity, and phagocytosis (Knödler et al., 2018).

Studies with this ADC have demonstrated its success as an immunotherapy (Fossati et al., 2015), leading to its approval by the European Medicines Agency (EMA) in 2009 for the intraperitoneal treatment of malignant ascites. However, the approval of this ADC was withdrawn in 2017 due to commercial reasons (Ruf et al., 2021).

#### 5.2.5 Abagovomab

The murine anti-idiotypic monoclonal antibody abagovomab was developed to functionally mimic the three-dimensional structure of CA-125 and induce a specific immune response directed against the original antigen (Battaglia et al., 2017). In this context, a phase III placebo-controlled study known as MIMOSA was conducted, but it showed that the survival rate of patients with OC was not increased by abagovomab (Battaglia et al., 2017). However, a study by Battaglia et al. (2017) aimed to demonstrate that a healthy immune system conditions the response to this ADC. In their research, 80 patients received abagovomab, and 31 patients received placebo. Patients treated with abagovomab who had a percentage of CD8^+^ T cells producing IFN-γ above the cutoff point showed better recurrence-free survival (*p* = 0.042) than those with a percentage of CD8^+^ T cells producing IFN-γ below the cutoff point. Additionally, this study demonstrated that the recurrence-free survival of patients treated with abagovomab with both a percentage of CD8^+^ T cells producing IFN-γ and absolute cell counts below the respective cutoff points was identical to that of patients in the placebo group. In this regard, it is concluded that further studies are needed to clarify the effects of abagovomab in OC patients.

#### 5.2.6 Daclizumab (Zenapax)

Daclizumab (Zenapax) is a humanized IgG1 monoclonal antibody specific to IL-2 receptor-α subunit (CD25) (Tse et al., 2014). It irreversibly blocks CD25, thereby preventing signaling through the high-affinity IL-2R while increasing the bioavailability of IL-2 to bind to the low-affinity receptor (Ranganath et al., 2020). As a result, ADC induces various immunological changes, including inhibition of T cell activation, reduction in the frequency and survival of regulatory T cells, and expansion of CD56bright NK cells (Ranganath et al., 2020).

Within this scenario, an interventional phase I clinical trial was conducted with patients with recurrent ovarian, fallopian tube, or primary peritoneal cancer using this ADC. However, the study was terminated in 2018, and the results were not published. Additionally, this drug was suspended by EMA in 2018 due to 12 reported worldwide cases of severe brain inflammation, three of which were fatal (European Medicines Agency, 2018). Table 2 highlights some studies that evaluate ICIs and ADCs technologies in OC treatment.

**TABLE 2:** Ongoing studies evaluating ICIs and ADCs technologies in OC.

Agents	Targets	Clinical study phase	Results	References
Nivolumab	PD-1	II	64.7% of patients experienced PFS at 24 weeks, and treatment toxicity was moderate	Friedman et al. (2024)
Ivonescimab (AKT112/SMT112)	PD-1/VEGF-A	Ia	Among 19 patients, 5 achieved a partial response, including 3 with high-grade serous pathology, resulting in an ORR of 26.3%. Furthermore, was observed that the disease remained stable for more than 12 months in 4 patients who had previously been treated with bevacizumab	Frentzas et al. (2024)
Ipilimumab + Nivolumab	CTLA-4 and PD-1	Ib and II	A partial response was observed in 2 patients, with a response duration of 14.8 months. In addition, 2 of 23 patients demonstrated adverse effects classified as grade 3 or higher	Knisely et al. (2024)
Durvalumab + Tremelimumab	PD-L1 and CTLA-4	II	There was no significant difference in PFS between the combination therapy group and the sequential therapy group In addition, partial responses were observed in 2 patients and stable disease in 1 patient, with all responses occurring in the combination therapy group	Hinchcliff et al. (2024)
Durvalumab + Eribulin	PD-L1 and microtubules	Ib	ORR of 55%, with 4 patients experiencing stable disease, and a PFS of 6.2 months	Landry et al. (2023)
Niraparib + Pembrolizumab	PARP and PD-1	I and II	ORR of 19%, compared to monotherapy with each agent, with no signs of toxicity	Konstantinopoulos et al. (2019)
JNJ-78306358	⍺3 domain of HLA-G and CD3	I	The therapy facilitated the formation of immune synapses and the killing of tumor cells by CTLs. Furthermore, no interaction of this ADC was found with cells that do not express HLA-G, demonstrating its specificity for certain types of tumor cells	Geva et al. (2024)
Mirvetuximab	FR⍺	III	Patients showed median PFS of 5.62 months and ORR of 42.3% During treatment, was demonstrating greater safety in relation to the group with the another treatment	Moore et al. (2023)
Mirvetuximab + Carboplatin + Bevacizumab	FR⍺ and VEGF	Ib	Patients showed an ORR of 83%. Most adverse effects were graded as 2 or lower	Richardson et al. (2024)
Oregovomab	CA-125	II	All patients achieved cytoreduction and the PFS demonstrating a significant difference between the control group and the treated group	Brewer et al. (2020) Junsik et al., 2024
Abagovomab	EpCAM and CD3	III	Patients showed better recurrence-free survival	Battaglia et al., 2017

### 5.3 T- and NK-cell engaging bispecific antibodies (BsAbs)

Bispecific antibodies (BsAbs) are engineered molecules designed to bind simultaneously to two distinct epitopes or antigens. This dual targeting mechanism allows them to interact with tumor antigens on cancer cells while activating receptors on immune cells, offering a novel approach to immunotherapy (Wang Q. et al., 2019). Recent studies have focused on the roles of T and NK cells in this context, as BsAbs can effectively bring these immune cells into proximity with tumor cells (Wu Z. and Cheung, 2018). By simultaneously binding to tumor antigens on cancer cells and activating receptors such as CD3 on T cells or CD16 on NK cells, BsAbs enhance the capacity of these immune cells to recognize and eliminate malignant cells. This strategy positions engaging BsAbs as a promising approach for cancer immunotherapy (Tapia-Galisteo et al., 2023).

In the context of hematological tumors, numerous clinical trials have demonstrated favorable outcomes with T cell-engaging bispecific antibodies (BsAbs). Notable examples include epcoritamab (Thieblemont et al., 2022), odronextamab (Bannerji et al., 2022), mosunetuzumab (Budde et al., 2022), and glofitamab (Hutchings et al., 2021). These CD3xCD20 T cell-engaging BsAbs bind to T cells via CD3 receptors, effectively directing them to eliminate malignant CD20^+^ B cells in patients with heavily pretreated B-cell non-Hodgkin lymphoma (van de Donk and Zweegman, 2023). Additionally, Reusing et al. (2021) reported that CD16xCD33 NK cell-engaging BsAbs activated Killer immunoglobulin-like receptor (KIR) signaling, thereby enhancing NK cell-mediated lysis of acute myeloid leukemia (AML) blasts.

Regarding solid tumors, particularly OC, Crawford and colleagues (2019) reported on the BsAb REGN4018, which targets both MUC16, a highly expressed marker in OC cells, and CD3, a receptor on T cells. Overall, their findings indicated that REGN4018 exhibited robust antitumor activity and favorable tolerability, warranting its clinical evaluation in patients with MUC16-expressing advanced OC (Crawford et al., 2019). Oladapo et al. (2021) similarly investigated T cell-engaging BsAbs targeting MUC16. Their findings indicate that these antibodies demonstrate efficacy against OC, both as a monotherapy and in combination with other agents such as PD-1 and VEGF inhibitors (Oladapo et al., 2021). In the other hand, Lee E. and colleagues (2021) examined a BsAb targeting LYPD1, an antigen associated with high-grade serous OC, and their data suggested its compelling efficacy and safety profiles, supporting its potential use as a treatment for high-grade serous OC (Lee E. et al., 2021).

Furthermore, Avanzino and colleagues (2022) studied TNB-928B, a T-cell engaging BsAb that binds to FRα to selectively target FRα overexpressing tumor cells. It was shown that TNB-928B induced preferential effector T-cell activation, proliferation, and selective cytotoxic activity on high FRα expressing OC cells, and also promoted T-cell infiltration and antitumor activity in OC mouse models (Avanzino et al., 2022). Additionally, Vallera et al. (2020) evaluated cam1615B7H3, a tri-specific killer engager that has a camelid CD16 antibody fragment, a wild-type IL-15 moiety, and an anti-B7-H3 single-chain variable fragment, in various types of solid tumors. Their findings suggest that cam1615B7H3 improves NK cell function, expansion, targeted cytotoxicity against various types of B7-H3-positive human cancer cell lines, and delivers an anti-cancer effect *in vivo* in a solid tumor setting, including in OC (Vallera et al., 2020).

Given the studies conducted, further research is necessary to ensure the safety of these ADCs in OC treatment.

## 6 Discussion

Overall, immunotherapy for OC faces significant challenges, yet the field holds substantial potential for advancement. Ongoing efforts aim to overcome immune suppression and improve the efficacy of OC immunotherapy. These strategies include combining immunotherapy with other drugs, utilizing targeted and precision-guided particles, developing innovative antigen vaccine delivery systems, and implementing prolonged low-dose immunotherapy regimens. Consequently, recent progress in both active and passive immunotherapy approaches has introduced new perspectives and insights, thereby enhancing the effectiveness of immune-based treatments for OC. Indeed, to handle the adverse effects of common therapies for cancer, immunotherapy strategies emerged as a cancer-specific alternative capable of targeting the tumor and causing minimal impact on normal tissues (Aly, 2012; Zhu and Yu, 2022). They are significant considering the usual therapeutic approaches such as surgery, chemotherapy, and radiotherapy which besides the adverse effects show a lack of specificity for tumors (Kaczmarek et al., 2023). Therapeutic cancer vaccination is a strategy of immunotherapy developed to elicit or boost antitumor adaptive immune responses to detect and eliminate them (Luo et al., 2024; Chambers, 2011). Moreover, CAR-T cells are genetically engineered to recognize and attack tumor-specific antigens (June et al., 2018), bypassing the need of MHC molecules presentation, and behaving as active drugs against tumors (Maus and June, 2016). In turn, exosomes are a category of extracellular vesicles with a lipid bilayer, measuring approximately 30–150 nm, found in various body fluids such as blood, urine, saliva, and cerebrospinal fluid (He et al., 2018; Gong et al., 2023). In addition to their ambiguous role, exosomes may serve as a potential tool for OC therapy (Shimizu et al., 2024). Also of clinical relevance, therapeutic monoclonal antibodies have been successfully developed for the treatment of various cancer types (Hafeez et al., 2020). Numerous clinical trials are still underway with promising prospects for the treatment of OC, including ADCs such as JNJ-78306358, ivonescimab, ipilimumab, durvalumab, oregovomab, catumaxomab, abagovomab, daclizumab and mirvetuximab, which was approved by the FDA in 2022 but remains under study for application in OC treatment (Dilawari et al., 2023). Yet, ICIs represent a promising class of drugs in immunotherapy against OC, targeting PD-1/PD-L1 and CTLA-4. They have already demonstrated broad bioactivity and stable response in the treatment of various types of tumors (Naimi et al., 2022; Tang S. et al., 2022b), including OC (Disis et al., 2019). Therefore, OC immunotherapy involves the induction of an anti-tumor immune response and the development of immunological memory. This process not only can eradicate malignant cells within the primary tumor site, thereby averting recurrence, but also hampers the metastatic spread to distant anatomical locations (Cha et al., 2020).
